# Future Challenges for the Diagnosis and Management of Affective Disorders: From Preclinical Evidence to Clinical Trials

**DOI:** 10.3390/brainsci15050489

**Published:** 2025-05-07

**Authors:** Mario Luciano, Antonio Ventriglio, Andrea Fiorillo

**Affiliations:** 1 Department of Psychiatry, University of Campania “Luigi Vanvitelli”, 80138 Naples, Italy; andrea.fiorillo@unicampania.it; 2 Department of Clinical and Experimental Medicine, University of Foggia, 71100 Foggia, Italy; dr.ventriglio@gmail.com

Affective Disorders (ADs) include a broad spectrum of clinical conditions, ranging from dysthymia and Major Depressive Disorder (MDD) to various forms of bipolar disorders (BD) and affective psychoses. The burden of these disorders is substantial at both the individual and familial levels, with significant implications for healthcare systems worldwide. ADs are not only associated with marked emotional distress and functional impairments but also with a substantial disruption in patients’ familial and social relationships [1,2]. Their high global burden reflects their prevalence, recurrent course, and the elevated mortality risk, with nearly 15% of individuals with MDD or BD dying by suicide [3].

According to the World Health Organization (WHO), MDD affects over 280 million people and is recognized as a leading cause of disability worldwide. BD, on the other hand, affects around 40 million individuals globally, with a severe impact on personal and professional functioning [4]. Beyond the individual burden, the economic impact of ADs is noteworthy, with considerable direct costs associated with treatment, hospitalizations, and long-term care. However, the indirect costs—including lost productivity, absenteeism, and disability claims—further exacerbate the financial strain on global economies. Research indicates that MDD alone costs the global economy over $1 trillion per year in lost productivity. Similarly, in the United States, the total economic burden of BD is estimated to exceed $45 billion annually, underscoring the far-reaching financial impact of these conditions beyond healthcare expenditures [5]. The economic burden is further compounded by the fact that many individuals remain undiagnosed or untreated, perpetuating a cycle of worsening symptoms and increased healthcare utilization [5].

The internalized stigma experienced by individuals with ADs is a major barrier to care, often leading to delays in seeking treatment due to fear of discrimination, exacerbation of symptoms, and reduced chances of full recovery [6]. In many low- and middle-income countries, mental health services remain underfunded and inaccessible to large segments of the population, further impeding effective management. Addressing stigma and enhancing mental health literacy through public health campaigns and educational initiatives are essential steps toward reducing the burden of ADs and minimizing the duration of untreated illness [7]. In this regard, delayed diagnoses and treatments are among the most critical factors contributing to poor outcomes in ADs. For many patients, it might take years before receiving an accurate diagnosis, leading to increased recurrence rates, greater severity of episodes, higher functional impairment, and higher risk of developing psychiatric and medical comorbidities [8]. For instance, individuals with bipolar disorder who were initially misdiagnosed as affected by MDD often receive antidepressant treatment, which may trigger manic or hypomanic episodes. Similarly, untreated depression can evolve into a chronic and treatment-resistant condition, significantly reducing the likelihood for clinical and personal recovery [7,8]. Prolonged delays in diagnosis are also associated with an elevated risk of suicide, as untreated individuals may experience worsening despair and hopelessness over time [6]. Improving screening, enhancing access to mental healthcare, and providing intervention as early as possible are crucial in order to mitigate the long-term impact of these disorders.

Another key factor shaping the clinical course of ADs is the high prevalence of psychiatric and medical comorbidities. In fact, individuals with mood disorders have an increased risk of developing cardiovascular disease, metabolic syndrome, immune-inflammatory dysregulation, and neurodegenerative disorders, among other conditions. Moreover, psychiatric comorbidities such as anxiety disorders, substance use disorders, and psychotic symptoms are frequent, leading to misdiagnoses and suboptimal treatment strategies. The bidirectional relationship between psychiatric and medical conditions suggests a shared pathophysiological basis, reinforcing the necessity for a holistic and integrative approach to diagnosis and treatment [9]. Current diagnostic criteria primarily rely on clinical observations and self-reported signs or symptoms, which are inherently subjective and susceptible to variability. In particular, the substantial symptomatic overlap between depression and bipolar disorder—especially during depressive episodes—frequently results in misdiagnosis and inappropriate treatment selection. A notable challenge is the frequent misclassification of bipolar depression as unipolar depression, leading to inappropriate antidepressant prescriptions that may exacerbate mood instability and increase the risk of manic episodes [10].

The Research Domain Criteria (RDoC) initiative, launched by the National Institute of Mental Health, represents a promising effort to move beyond traditional diagnostic categories by integrating behavioral, cognitive, and neurobiological markers [11]. Advances in neuroscience and biomarker research have provided compelling evidence for improving the diagnostic accuracy of ADs [12]. Emerging findings indicate that mood disorders are characterized by significant alterations in inflammatory markers, oxidative stress levels, and metabolic dysregulation [13]. Additionally, neuroimaging and genetic studies have identified distinct neurobiological and genetic features, reinforcing the potential of biomarker-based approaches in clinical practice. The increasing use of machine-learning algorithms and computational psychiatry models allows for the integration of large-scale datasets encompassing clinical, neurobiological, and genetic information, facilitating improved diagnostic precision and personalized treatment strategies [14]. Furthermore, precision medicine approaches incorporating biosignatures hold promise for optimizing therapeutic outcomes, reducing trial-and-error prescribing, and enhancing long-term prognosis.

Beyond biomarkers, novel therapeutic approaches are emerging in the treatment of ADs. Neuromodulation techniques, including Transcranial Magnetic Stimulation (TMS) and Deep Brain Stimulation (DBS), have demonstrated efficacy in treatment-resistant affective disorders. Additionally, psychedelic-assisted therapy—particularly with compounds such as psilocybin and ketamine—is gaining increasing empirical support for its rapid-acting antidepressant effects. Meanwhile, evidence-based psychosocial interventions, including cognitive-behavioral therapy (CBT), interpersonal therapy (IPT), and psychoeducational programs, have demonstrated significant efficacy in reducing relapse rates and improving overall functioning [15].

This Special Issue, “Future Challenges for the Diagnosis and Management of Affective Disorders: From Preclinical Evidence to Clinical Trials”, seeks to bridge the gap between preclinical research and clinical applications. By exploring novel diagnostic tools, biomarker-based approaches, and innovative therapeutic strategies, this collection provides up-to-date insights into the complexities of ADs and potential pathways for improving their management. Contributions in this issue address the multifaceted nature of these disorders from interdisciplinary perspectives. In this context, authors illustrate how the integration of biological, psychological, and social factors remains fundamental to understanding the heterogeneity of ADs and optimizing their management.

## References

[1] von Kardorff E., Soltaninejad A., Kamali M., Eslami Shahrbabaki M. (2016). Family caregiver burden in mental illnesses: The case of affective disorders and schizophrenia—A qualitative exploratory study. Nord. J. Psychiatry.

[2] Di Vincenzo M., Sampogna G., Della Rocca B., Brandi C., Mancuso E., Landolfi L., Volpicelli A., Di Cerbo A., Fiorillo A., Luciano M. (2022). What influences psychological functioning in patients with mood disorders? The role of clinical, sociodemographic, and temperamental characteristics in a naturalistic study. Ann. Gen. Psychiatry.

[3] Hayes S.C., Pistorello J. (2024). Can a practical process-oriented strategy prevent suicidal ideation and behavior?. World Psychiatry.

[4] World Health Organization (WHO) Mental Health Atlas 2020. https://www.who.int/publications/i/item/9789240036703.

[5] Arias D., Saxena S., Verguet S. (2022). Quantifying the global burden of mental disorders and their economic value. eClinicalMedicine.

[6] Fabrazzo M., Cipolla S., Pisaturo M., Camerlengo A., Bucci P., Pezzella P., Coppola N., Galderisi S. (2023). Bidirectional Relationship between HIV/HBV Infection and Comorbid Depression and/or Anxiety: A Systematic Review on Shared Biological Mechanisms. J. Pers. Med..

[7] Falkai P., Möller H.J. (2010). Affective disorders: The role of the duration of untreated illness, suicidality and pharmacogenetics. Eur. Arch. Psychiatry Clin. Neurosci..

[8] Colomer L., Anmella G., Vieta E., Grande I. (2021). Physical health in affective disorders: A narrative review of the literature. Rev. Bras. Psiquiatr..

[9] Berk M., Köhler-Forsberg O., Turner M., Penninx B.W.J.H., Wrobel A., Firth J., Loughman A., Reavley N.J., McGrath J.J., Momen N.C. (2023). Comorbidity between major depressive disorder and physical diseases: A comprehensive review of epidemiology, mechanisms and management. World Psychiatry.

[10] Stahl S.M., Morrissette D.A., Faedda G., Fava M., Goldberg J.F., Keck P.E., Lee Y., Malhi G., Marangoni C., McElroy S.L. (2017). Guidelines for the recognition and management of mixed depression. CNS Spectr..

[11] Cuthbert B.N. (2020). The role of RDoC in future classification of mental disorders. Dialogues Clin. Neurosci..

[12] Berk M. (2023). Biomarkers in psychiatric disorders: Status quo, impediments and facilitators. World Psychiatry.

[13] Abi-Dargham A., Moeller S.J., Ali F., DeLorenzo C., Domschke K., Horga G., Jutla A., Kotov R., Paulus M.P., Rubio J.M. (2023). Candidate biomarkers in psychiatric disorders: State of the field. World Psychiatry.

[14] Ricci F., Giallanella D., Gaggiano C., Torales J., Castaldelli-Maia J.M., Liebrenz M., Bener A., Ventriglio A. (2025). Artificial intelligence in the detection and treatment of depressive disorders: A narrative review of literature. Int. Rev. Psychiatry.

[15] Marwaha S., Palmer E., Suppes T., Cons E., Young A.H., Upthegrove R. (2023). Novel and emerging treatments for major depression. Lancet.

